# Short-chain fatty acids as key mediators of the microbiota–gut–brain axis in chronic cerebral hypoperfusion: mechanisms and therapeutics

**DOI:** 10.1186/s10020-026-01616-z

**Published:** 2026-08-27

**Authors:** Junchen Si, Peijian Wang, Zhongchen Li, Jiheng Hao, Jiyue Wang, Liyong Zhang

**Affiliations:** Department of Neurosurgery, Liaocheng People’s Hospital, NO.45 Huashan Road, Liaocheng, Shandong 252000 China

**Keywords:** Chronic cerebral hypoperfusion, Short-chain fatty acids, Microbiota dysbiosis, Neuroinflammation, Cognitive impairment

## Abstract

Chronic cerebral hypoperfusion (CCH) is an important contributor to vascular cognitive impairment and neurodegenerative disorders, characterized by neuronal injury, blood–brain barrier (BBB) disruption, neuroinflammation, mitochondrial dysfunction, and synaptic impairment. Increasing evidence suggests that the microbiota–gut–brain axis plays a crucial role in ischemic brain injury. CCH is associated with gut microbiota dysbiosis, intestinal barrier dysfunction, and reduced production of microbiota-derived short-chain fatty acids (SCFAs), which may be linked to cognitive decline through bidirectional gut–brain communication. This review summarizes the mechanisms potentially involved in CCH-related neurological damage and highlights the potential neuroprotective effects of SCFAs, including regulation of neuroinflammation, BBB integrity, mitochondrial homeostasis, neuronal apoptosis, and synaptic plasticity. Furthermore, potential microbiota-targeted therapeutic strategies, such as fecal microbiota transplantation, probiotics/prebiotics, and SCFA supplementation, are discussed. Targeting gut microbiota-derived SCFAs may represents a promising approach for improving CCH-associated cognitive impairment, although further clinical validation is required.

Chronic cerebral hypoperfusion (CCH) is a pathological condition characterized by a sustained 20%–40% reduction in cerebral blood flow (CBF), which serves as a critical contributor to the onset and progression of vascular cognitive impairment and neurodegenerative diseases (Poh et al. 2022; Ciacciarelli et al. 2020). CCH may involve widespread cerebral regions or selectively affect vulnerable brain areas. Persistent cerebral hypoperfusion disrupts blood–brain barrier (BBB) integrity and accelerates white matter degeneration, resulting in demyelination and axonal loss within the central nervous system. These pathological processes contribute to progressive cognitive deterioration and increase the risk of developing vascular dementia (VaD) and Alzheimer’s disease (AD) (Duncombe et al. 2017; Rajeev et al. 2022; Zheng et al. 2019). VaD accounts for approximately 15% to 20% of clinically diagnosed dementia cases in North America and Europe, with this proportion rising to nearly 30% in Asia and several developing countries (Gorelick et al. 2011; Chan et al. 1990; Jhoo et al. 2008). It is estimated that approximately 7.7 million new cases of dementia occur annually,thus,CCH poses a significant threat to both human health and quality of life (Iadecola 2013).

In recent years, the association between alterations in the gut microbiota and neurological disorders has emerged as a prominent area of research. Increasing evidence supports bidirectional communication between the gut and the brain (Lee et al. 2020; Bo et al. 2023; Nazarova et al. 2022). CCH not only contributes to cognitive impairment by inducing neuronal apoptosis but also disrupts the gut microbiota through the brain–gut axis, leading to gut dysbiosis, impaired intestinal barrier function, and reduced production of short-chain fatty acids (SCFAs) (Xiao et al. 2022). Conversely, gut microbial dysbiosis and alterations in microbiota-derived metabolites may exacerbate CCH-related neurological injury through the microbiota–gut–brain axis (Singh et al. 2016). This reciprocal interaction between the brain and the gut provides novel opportunities for the prevention and treatment of CCH-associated cognitive dysfunction. This review summarizes the mechanisms underlying CCH-induced neurological injury, bidirectional communication along the microbiota–gut–brain axis, and potential therapeutic strategies for restoring gut microbial homeostasis, with the aim of providing insights that may inform future clinical interventions.

## Mechanisms of central nervous system injury induced by CCH

**Fig. 1:**
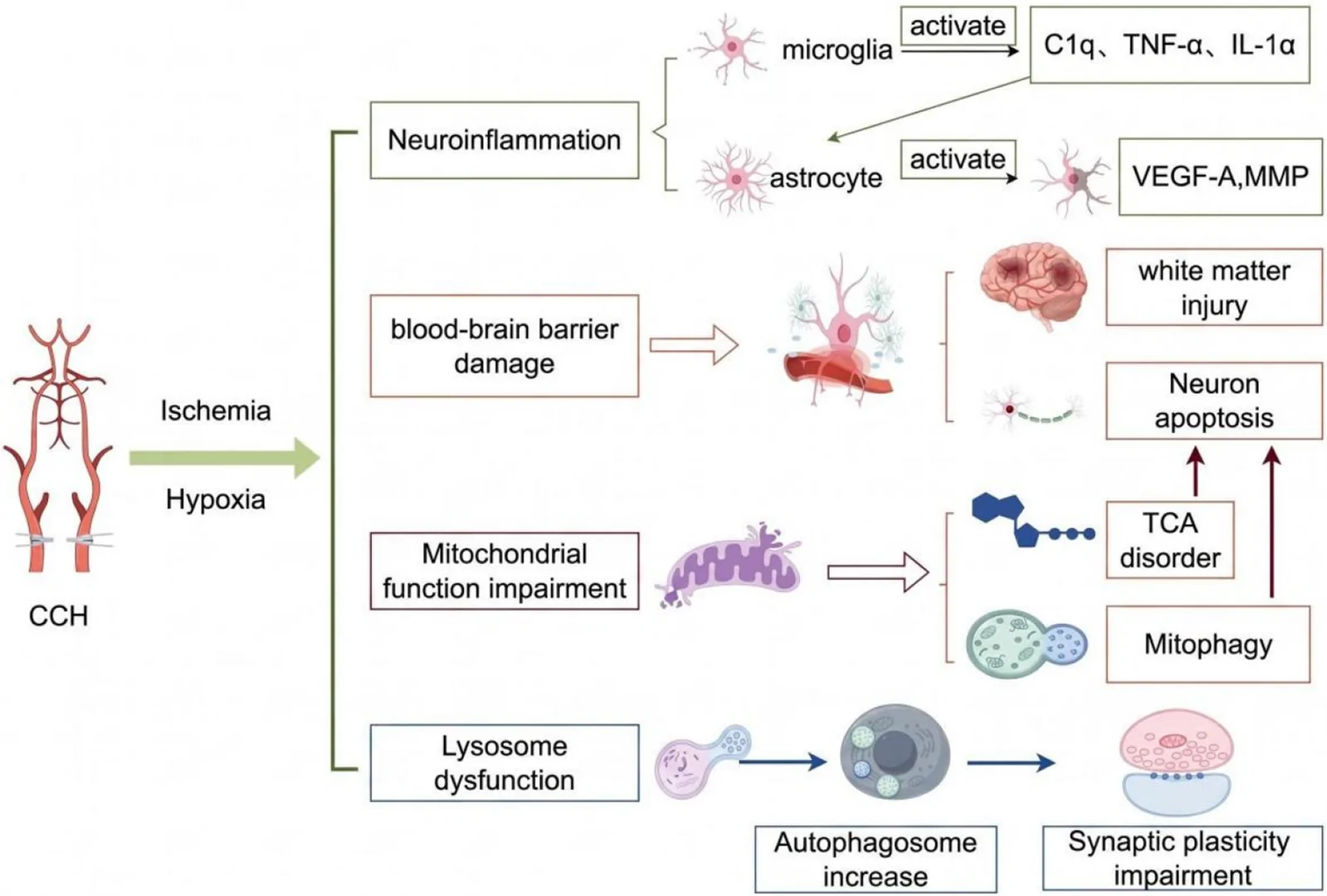
Central nervous system injury induced by chronic cerebral hypoperfusion (This figure was drawn by Figdraw: UOOOA6ae74)

CCH has been associated with multiple pathological alterations in the brain, such as neuroinflammation (Zhao et al. 2021), metabolic disturbances (Daulatzai 2016), neuronal apoptosis (Poh et al. 2020), oxidative stress damage (Hei et al. 2018), BBB disruption (Huang et al. 2020), synaptic plasticity impairment (<aging-13–202555.pdf> n.d.), and mitochondrial damage (Tang 2016) (Fig. 1). Among these interconnected pathological processes, neuroinflammation and mitochondrial dysfunction have been identified as important contributors to CCH-induced cognitive impairment (Du et al. 2016). While other mechanisms also participate in cognitive impairment development.

### Neuroinflammation

In the central nervous system (CNS), microglia and astrocytes are actively involved in immune and inflammatory responses. Microglia function as resident phagocytes in the CNS, clearing apoptotic cells and cellular debris and participating in synaptic remodeling (Waisman et al. 2015). Astrocytes contribute to nearly all aspects of CNS function, including synapse formation and maturation, ion and neurotransmitter homeostasis, neurodevelopment, blood–brain barrier (BBB) maintenance, and metabolic support for neurons (Waisman et al. 2015; Miyamoto et al. 2020). CCH can activate microglia, which subsequently release pro-inflammatory mediators, including complement component 1q (C1q), tumor necrosis factor-α (TNF-α), and interleukin-1α (IL-1α). These mediators can act on the cerebral white matter, thereby contributing to CNS injury and neuronal loss (Duncombe et al. 2017). Elevated levels of inflammatory mediators may also induce BBB disruption, neuronal apoptosis, and cognitive dysfunction, including memory impairment (Hickman et al. 2018; Mirzaei et al. 2021). Pro-inflammatory mediators released by microglia in response to CCH can induce the transition of astrocytes from a supportive and neuroprotective phenotype to a neurotoxic reactive phenotype (Kopp et al. 2022). These reactive astrocytes may damage neurons and oligodendrocytes by releasing apolipoprotein E (APOE) and saturated lipids carried by apolipoprotein J (APOJ)-containing lipoprotein particles (Liddelow et al. 2017). In addition, during ischemic stroke, astrocytes secrete multiple inflammatory mediators, including vascular endothelial growth factor A (VEGF-A), matrix metalloproteinases (MMPs), and chemokines (Zhang et al. 2023). Increased levels of these mediators may directly or indirectly aggravate cerebral injury and compromise BBB integrity, thereby promoting further neurological damage.

### Blood–brain barrier damage

BBB plays a critical role in maintaining central nervous system homeostasis and regulating neuroimmune responses.Its principal functions include preserving a stable cerebral microenvironment, restricting the entry of circulating toxins and pathogens, and protecting the brain from infection (Rajeev et al. 2022). Cerebral ischemia can induce oxidative stress in brain tissue, resulting in excessive production of reactive oxygen species (ROS). These reactive species directly damage BBB endothelial cells and contribute to increased vascular permeability and vasogenic edema (Candelario-Jalil et al. 2022). Matrix metalloproteinase-9 (MMP-9) plays a key role in BBB disruption following ischemic stroke by degrading tight junction proteins and thereby compromising barrier integrity (Reeson et al. 2015; Rempe et al. 2016). BBB disruption can initiate a cascade of pathological changes, leading to synaptic dysfunction, neuronal injury, and cognitive impairment (Valdes Hernandez et al. 2017). It has been reported that BBB permeability is significantly increased in patients with VaD (Farrall and Wardlaw 2009), and that neuronal loss and white matter degeneration accompany the progression of the disease (Toth et al. 2017). A study on CCH in rodents highlighted the critical role of BBB dysfunction in the development of white matter injury. BBB disruption may represent an early event in white matter lesion formation, and increased BBB permeability has been associated with the severity of white matter pathology (Rajeev et al. 2022; Huang et al. 2018). Collectively, these findings indicate a close pathological relationship between BBB dysfunction and white matter injury. CCH may increase BBB permeability and thereby aggravate white matter damage, potentially contributing to the development and progression of stroke-related neurological dysfunction and VaD.

### Mitochondrial dysfunction

Mitochondria, as regulators of cellular energy homeostasis and cell death signaling, are crucial for the physiological and biochemical activities of neuron (Mayevsky et al. 2020). Mitochondrial dysfunction has been identified as an important pathological mechanism underlying ischemia-induced neuronal apoptosis (Anzell et al. 2018). Studies have shown that CCH can inhibit the activity of mitochondrial electron transport chain complexes I-V, thereby reducing ATP production and ultimately leading to impair mitochondrial bioenergetic function (Su et al. 2022). In addition to affecting ATP synthesis, CCH can induce mitochondrial membrane depolarization, increase mitochondrial membrane permeability and reactive oxygen species (ROS) production, and elevate intracellular Ca^2^⁺ levels, thereby activating the intrinsic apoptotic pathway (Yu et al. 2023). These factors collectively contribute to mitochondrial damage, resulting in increased neuronal apoptosis and exacerbating neurological dysfunction.

**Fig. 2:**
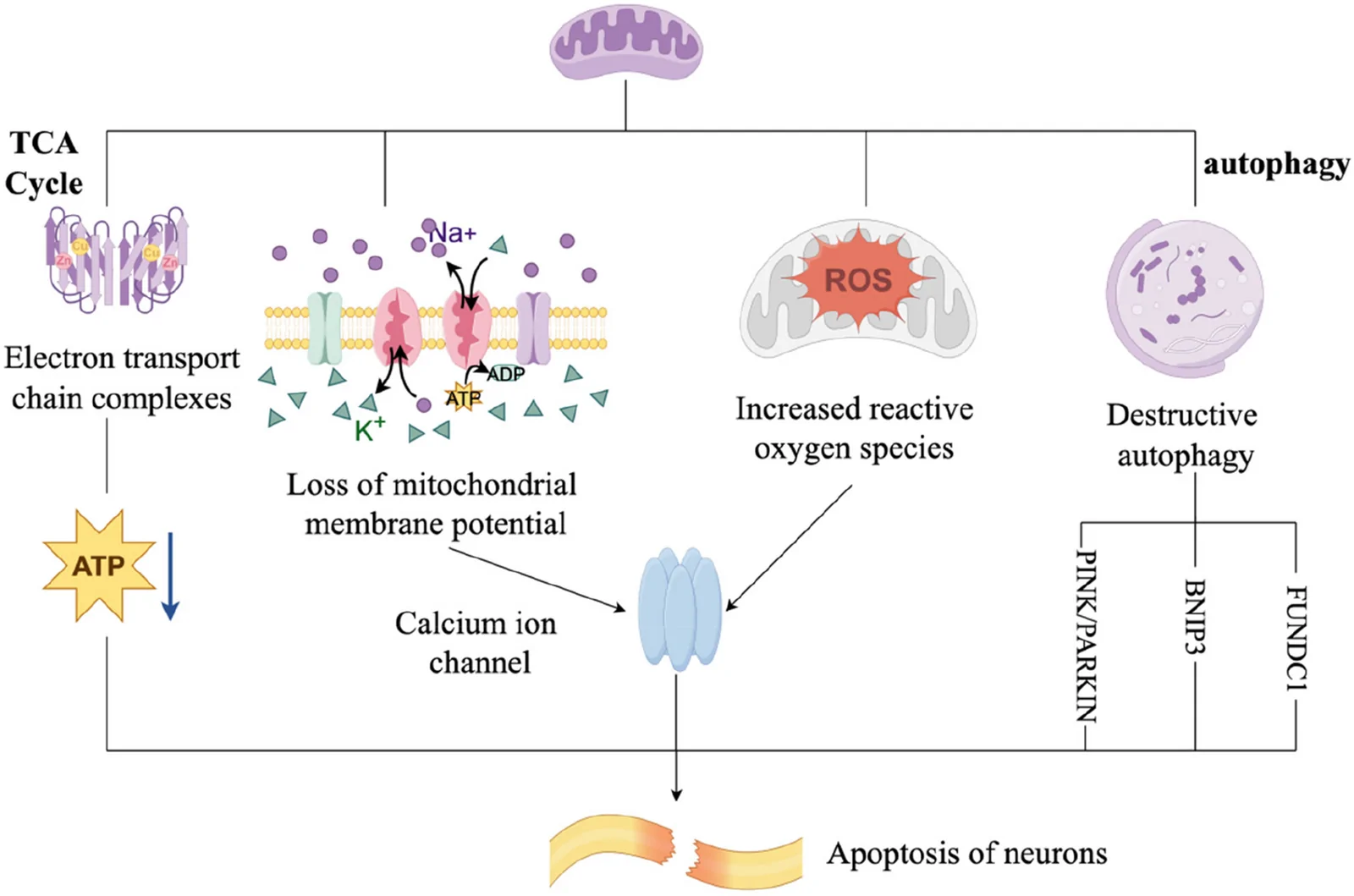
Effects of chronic cerebral hypoperfusion on mitophagy (This figure was drawn by Figdraw: SRUUI6e6a6)

Neuronal survival and apoptosis are also modulated by mitophagy, a selective form of autophagy that removes damaged mitochondria. Mitophagy has dual functions: protective autophagy and destructive autophagy (Zhang et al. 2022). Protective autophagy refers to the maintenance of neuronal homeostasis through low-level autophagy, whereas excessive autophagy may lead to neuronal loss, hippocampal atrophy, and synaptic disintegration, contributing to the onset and progression of cerebrovascular diseases and cognitive dysfunction (Su et al. 2018; Wang et al. 2020). The PTEN-induced kinase 1 (PINK1)/Parkin pathway is a major signaling pathway involved in the regulation of mitophagy. Evidence suggests that ROS generated during chronic cerebral ischemia may activate PINK1/Parkin-mediated mitophagy (Wei et al. 2015). BCL2/adenovirus E1B 19-kDa-interacting protein 3 (BNIP3), which is expressed in multiple cell types, is localized to the outer mitochondrial membrane and promotes the recruitment of autophagosomes by interacting with microtubule-associated protein 1 light chain 3 (LC3), thereby mediating mitophagy (Tian et al. 2022). Phosphorylation of BNIP3 may enhance its interaction with LC3 and promote mitophagy under hypoxic conditions. Dysregulated BNIP3-mediated mitophagy may therefore influence the severity of ischemic brain injury, suggesting that this pathway may represent a potential therapeutic target for ischemic stroke (Yuan et al. 2017). FUN14 domain-containing protein 1 (FUNDC1) is another outer mitochondrial membrane mitophagy receptor that interacts with LC3 and regulates mitochondrial quality control under hypoxic and ischemic conditions (Jeanneret and Yepes 2017; Cai et al. 2021) (Fig 2). Existing evidence suggests that the BNIP3- and PINK1/Parkin-mediated pathways may be involved in mitophagy induced by CCH (Su et al. 2018; Li et al. 2025). Although FUNDC1-mediated mitophagy has received increasing attention, direct evidence regarding its involvement in CCH-induced mitophagy remains limited. Further studies are therefore required to determine whether FUNDC1 contributes to CCH-related mitochondrial dysfunction. In conclusion, mitophagy regulates neuronal survival and apoptosis through multiple signaling pathways and may provide potential therapeutic targets for CCH.

### Synaptic plasticity damage

Studies have shown that in the BCCAO rat model, CCH leads to damage in synaptic plasticity and basal synaptic transmission, resulting in impaired hippocampal long-term potentiation and memory decline, ultimately progressing to VaD (Sharifi et al. 2021; Damodaran et al. 2019). CCH has also been shown to reduce synaptophysin expression in the hippocampal CA1 region, induce lysosomal dysfunction, promote autophagosome accumulation, and trigger excessive autophagy, ultimately impairing synaptic plasticity and cognitive function (Su et al. 2016, 2018). In addition, CCH reduces the phosphorylation of calcium/calmodulin-dependent protein kinase II (CaMKII), and this reduction suppresses LTP and compromises synaptic transmission and plasticity (Li et al. 2017). Collectively, these findings indicate that CCH impairs synaptic plasticity, thereby contributing to learning and memory deficits and broader cognitive dysfunction.

## CCH and gut microbiota dysbiosis

The human microbiota comprises diverse communities of microorganisms that colonize various body sites and mucosal surfaces, including the oral and nasal cavities, respiratory tract, reproductive tract, and gastrointestinal tract. Approximately 90% of these microbiota are found in the initial parts of the small and large intestines (Muhammad et al. 2023; Goralczyk-Binkowska et al. 2022). The majority of gut bacteria belong to the phyla Bacteroidetes and Firmicutes (comprising approximately 51% and 48%, respectively), while other microbial groups, such as Actinobacteria (including Bifidobacterium), Spirochaetes, and Proteobacteria, are present at lower abundances (Chidambaram et al. 2022). The gut microbiota performs several essential functions. First, it contributes to the maintenance of intestinal homeostasis by modulating luminal pH, intestinal motility, and bowel function (Goralczyk-Binkowska et al. 2022). Second, the gut microbiota produces short-chain fatty acids (SCFAs) through the anaerobic fermentation of dietary fiber. These metabolites, particularly butyrate, serve as major energy sources for colonic epithelial cells and support intestinal barrier integrity (Silva et al. 2020). Third, another important function of the gut microbiota is to neutralize toxins and carcinogenic compounds (Claus et al. 2016). In summary, the gut microbiota plays an important role in maintaining physiological homeostasis, and its bidirectional interactions with multiple organs have attracted increasing research attention.

**Fig. 3:**
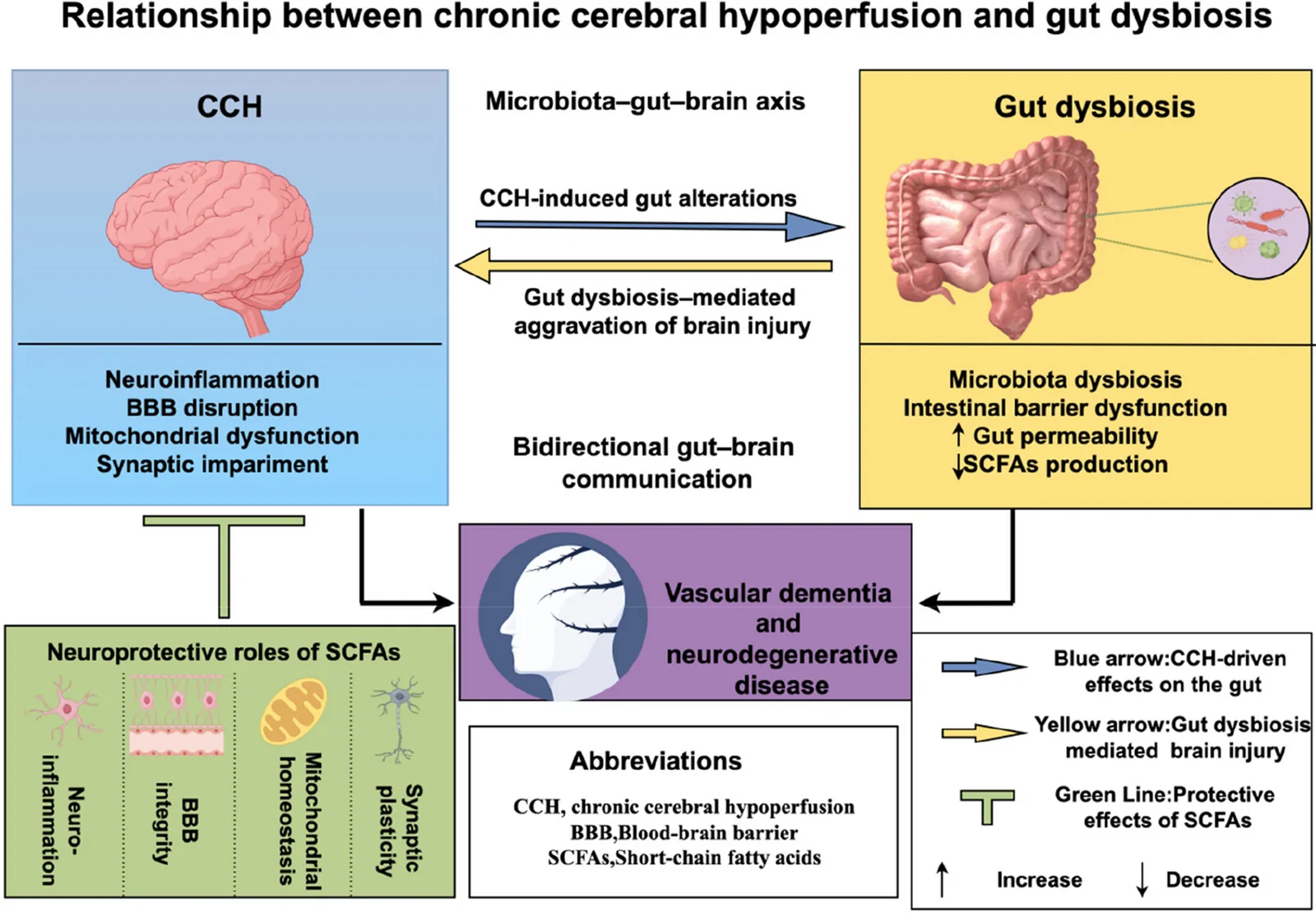
Relationship between chronic cerebral hypoperfusion and gut dysbiosis (This figure was drawn by Figdraw: WOYUT73479)

The central nervous system, gut microbiota, and gastrointestinal tract form a bidirectional communication network known as the microbiota–gut–brain axis (MGBA) (Zhang et al. 2024). The association between CCH and gut microbial dysbiosis has received increasing attention (Fig. 3). However, the mechanisms by which CCH disrupts intestinal microbial homeostasis remain incompletely understood and are likely multifactorial. Experimental evidence indicates that CCH is accompanied by gut microbial dysbiosis, disruption of the intestinal epithelial barrier, impaired mucosal immune homeostasis, and altered colonic expression of immune- and inflammation-related cytokines, including tumor necrosis factor-α (TNF-α), interleukin-1β (IL-1β), IL-6, IL-4, and IL-10 (Xiao et al. 2022). These findings suggest that chronic hypoperfusion may disrupt both epithelial and immune homeostasis in the intestine. CCH has also been shown to reduce the relative abundance of SCFA-associated genera, including Romboutsia, Turicibacter, and Prevotella, and to decrease the production of short-chain fatty acids (SCFAs), particularly acetate and propionate, thereby compromising colonic barrier integrity and promoting intestinal inflammation (Su et al. 2022). Rats with vascular dementia exhibit alterations in the relative abundance of Firmicutes and Patescibacteria, accompanied by reduced goblet-cell numbers and mucus secretion, increased inflammatory-cell infiltration, and decreased expression of the tight-junction proteins occludin and ZO-1 (Ren et al. 2025). These alterations may partly result from hypoxic and metabolic stress and impaired microvascular perfusion in the intestinal mucosa, which may disrupt epithelial energy metabolism and exacerbate intestinal inflammatory responses. CCH-related brain injury may impair autonomic regulation and enteric nervous system function, thereby altering intestinal motility, secretion, mucus production, and the epithelial microenvironment. For example, vasoactive intestinal peptide-mediated signaling has been implicated in the regulation of intestinal epithelial and goblet cell function (Prame Kumar et al. 2025). Persistent ischemic stress may further activate neuroendocrine stress responses and alter glucocorticoid signaling. These changes may affect the expression and distribution of tight junction proteins, such as occludin and claudins, increase paracellular permeability, and promote the translocation of bacteria and microbial products (Zong et al. 2019). Collectively, these findings suggest that vascular and metabolic stress, autonomic and neuroendocrine dysfunction, epithelial-barrier impairment, and alterations in the intestinal immune microenvironment interact through the brain–gut axis, forming a self-reinforcing cycle that drives and sustains gut dysbiosis during CCH.

Gut microbiota dysbiosis can also influence CCH through the MGBA. Emerging evidence suggests that the gut microbiota plays an important role in maintaining BBB integrity. Compared with conventionally colonized mice, germ-free mice exhibit increased BBB permeability accompanied by cognitive impairment (Zemmel et al. 2025). A subsequent study further demonstrated that germ-free mice exhibited increased BBB permeability beginning during intrauterine development and persisting into adulthood, which was associated with reduced expression of the tight junction proteins occludin and claudin-5 (Braniste et al. 2014). Gut microbiota dysbiosis may also impair mitochondrial metabolism, thereby contributing to neuronal apoptosis. During intestinal inflammation, excessive production of reactive oxygen species (ROS) can impair mitochondrial function and trigger apoptotic pathways (Haque et al. 2024). Gut microbiota dysbiosis may further disrupt mitochondrial respiration by reducing microbial folate production and may alter nervous system function through activation of the mechanistic target of rapamycin (mTOR) signaling pathway (Silva et al. 2017). Another study found that the metabolic product of gut microbiota, trimethylamine N-oxide (TMAO), increased the number of senescent cells (mainly neurons), enhanced mitochondrial damage, and elevated superoxide production, thus promoting brain aging in mice (Li et al. 2018). These findings indicate that gut microbiota dysbiosis may impair mitochondrial function in brain tissue through the microbiota–gut–brain axis, ultimately contributing to neuronal dysfunction. Conversely, the gut microbiota can influence and regulate brain development and function by altering microbial substrates, fermentation products, and gut-derived endocrine signals. Modulation or restoration of the gut microbiota may therefore help ameliorate cognitive impairment associated with CCH. Studies have shown that fecal microbiota transplantation(FMT) improves gut microbial composition, attenuates neuronal injury, and alleviates cognitive deficits in rats with CCH (Xiao et al. 2022; Su et al. 2022). Other studies have also demonstrated that correcting gut microbiota dysbiosis through dietary or pharmacological interventions may mitigate CCH-induced cognitive impairment (Bo et al. 2023; Song et al. 2024). Taken together, CCH disrupts the gut microbiota and intestinal homeostasis through brain-to-gut signaling, whereas interventions targeting the gut microbiota may alleviate CCH-associated cognitive impairment through gut-to-brain signaling, highlighting the bidirectional regulatory relationship between the brain and the gut.

## The role of SCFAs in CCH

SCFAs, the most important metabolic products of gut bacteria, primarily include of acetate, propionate, and butyrate, and are produced by the fermentation of dietary fibers by the gut microbiota (Su et al. 2022). SCFAs play important roles in regulating immune function, maintaining nutritional and metabolic homeostasis, and supporting the development and maturation of the nervous system (Henry et al. 2021; Erny et al. 2015). SCFAs are key metabolic products of gut microbiota, exhibiting diverse types, origins, and functions in brain metabolism (Table 1). Acetate, propionate, and butyrate appear to exert distinct yet complementary effects in CCH. Acetate has recently been shown to ameliorate cognitive deficits in mice subjected to bilateral common carotid artery stenosis, potentially through modulation of the FFAR3/ERK signaling pathway (Liu et al. 2025). In addition, acetate may contribute to the restoration of intestinal barrier integrity, Treg/Th17 homeostasis, and mitochondrial oxidative phosphorylation (Su et al. 2023). In contrast, the independent role of propionate in CCH remains unclear, as the available evidence is primarily derived from studies involving combined SCFA supplementation. Butyrate has demonstrated direct cognitive benefits in rat models of chronic cerebral hypoperfusion (Liu et al. 2015). Butyrate, as an important component of SCFA, protects neural function by reducing abnormal microglial activation and neuroinflammatory responses (Dalile et al. 2019; Wei et al. 2023).

**Table 1:** The types of Short-Chain Fatty Acids (SCFAs), their corresponding gut microbiota, and the role of SCFAs in brain metabolism (Ju et al. 2023; Ziętek et al. 2021)

SCFA	construct	Gut Microbiota	Role in Brain Metabolism
Acetate	C2	Bifidobacterium, Lactobacillus, Akkermansia, Prevotella and Ruminococcus	Crosses the blood–brain barrier (BBB) and serves as a substrate for acetyl-CoA, which is involved in neurotransmitter synthesis and brain energy metabolism
Propionate	C3	Prevotella, Veillonella, Bacteroides and Lachnospiraceae	Influences brain function by serving as a precursor for gluconeogenesis in the liver, affecting glucose levels, which are critical for brain energy
Butyrate	C4	Lachnospiraceae, Ruminococcaceae and Roseburia	Acts as a histone deacetylase inhibitor (HDACi), influencing gene expression, reducing neuroinflammation, and potentially improving cognitive function
Valeric acid	C5	Bacteroides and Clostridium	extremely rare,potentially influences neurotransmitter synthesis and neuroinflammation
caproic acid	C6	Bacteroides and Clostridium	potentially influences neurotransmitter synthesis and neuroinflammation
Isobutyrate	C4	Bacteroides and Clostridium	Less well-studied in brain metabolism; primarily involved in protein metabolism, potential influence on gut-brain axis via microbial metabolites
Isovalerate	C5	Bacteroides and Clostridium	Similar to isobutyrate; potential indirect effects on brain function through modulation of the gut microbiota composition and metabolic products

As major signaling mediators of the microbiota–gut–brain axis, SCFAs contribute to the maintenance of intestinal barrier integrity and regulate immune and inflammatory responses (Salminen 2023). SCFAs can cross the BBB via monocarboxylate transporters and may also modulate neural function indirectly through immune, endocrine, vagal, and humoral pathways, thereby influencing cognition, learning, memory, and emotion (Dalile et al. 2019; Mitchell et al. 2011). Increasing evidence indicates that SCFAs are involved in several central nervous system disorders, including Alzheimer’s disease, Parkinson’s disease, and post-stroke cognitive impairment, and may affect neurological recovery after stroke (Russo et al. 2018; Liu et al. 2020, 2022). A human study found that individuals at high risk of stroke exhibited a lower abundance of butyrate-producing bacteria and reduced fecal butyrate concentrations (Zeng et al. 2019). Our previous research has confirmed that CCH rats exhibit gut microbiota dysbiosis and a reduction in SCFA levels in the hippocampus compared to normal rats; cognitive function in CCH rats was significantly improved after intervention with gut microbiota modulation or direct SCFA supplementation (Xiao et al. 2022). Under chronic cerebral hypoperfusion conditions, impaired SCFA production and metabolism have been associated with intestinal barrier dysfunction, neuroinflammation, neuronal apoptosis, and cognitive impairment (Xiao et al. 2022; Su et al. 2023; Cheng et al. 2024).

### SCFAs and their receptors

SCFAs function as endogenous ligands for G protein-coupled receptors (GPCRs), thereby activating downstream signalling pathways involved in the regulation of inflammation, energy metabolism, and intestinal homeostasis. Among these receptors, GPR41, also known as free fatty acid receptor 3 (FFAR3), and GPR43, also known as free fatty acid receptor 2 (FFAR2), are the principal SCFA-sensing receptors. Their distinct ligand preferences and tissue distribution contribute to their differential metabolic and immunomodulatory effects (Parada Venegas et al. 2019). GPR109A, also known as hydroxycarboxylic acid receptor 2 (HCAR2), is predominantly expressed in intestinal epithelial cells, macrophages, microglia, and its expression is regulated by the gut microbiota (Sivaprakasam et al. 2016).

GPR41 is preferentially activated by propionate and butyrate. SCFA–GPR41 signalling contributes to microbiota–gut–brain communication and may influence brain physiology by regulating hormone secretion, intestinal motility, autonomic nervous system activity, and metabolic homeostasis (Wang et al. 2025; Kimura et al. 2011; Yang et al. 2022). Emerging evidence further suggests that GPR41-mediated signalling may be involved in the maintenance of mitochondrial homeostasis and BBB integrity (Yang et al. 2022).The SCFA receptor GPR43, also known as free fatty acid receptor 2 (FFAR2), is predominantly expressed in intestinal epithelial cells and various immune cells, including neutrophils, monocytes, macrophages, and dendritic cells. Activation of GPR43 regulates intracellular Ca^2^⁺ signalling, leukocyte chemotaxis, cytokine production, and the regulation and resolution of inflammatory responses. Therefore, the immunomodulatory effects of acetate and propionate may be mediated, at least partly, through GPR43-dependent pathways that maintain peripheral immune homeostasis and intestinal barrier integrity, thereby contributing to the regulation of neuroinflammation via the gut–brain axis (Le Poul et al. 2003; Maslowski et al. 2009).

Among the major SCFAs, butyrate is the principal endogenous agonist of GPR109A and mediates anti-inflammatory signalling, maintenance of intestinal barrier integrity, and mucosal immune tolerance, thereby contributing to intestinal homeostasis (Yu et al. 2024; Priyadarshini et al. 2018).In experimental models of neuroinflammation, butyrate-induced GPR109A activation promotes anti-inflammatory phenotypes in macrophages and dendritic cells, enhances interleukin-10-producing T-cell responses and regulatory T-cell differentiation, and modulates microglial activation, collectively contributing to the regulation of neuroinflammatory processes (Singh et al. 2014; Moutinho et al. 2022). However, whether GPR109A-mediated signalling directly contributes to neuroprotection in CCH remains to be further elucidated.

### SCFAs inhibit neuroinflammation

Alterations in the gut microbiota of patients with stroke are associated with decrease in SCFA levels, which may influence post-stroke neuroinflammation and T-cell differentiation (Henry et al. 2021). The increase in gut SCFA levels facilitated the repair of the damaged intestinal barrier after BCCAO surgery and inhibited the production of inflammatory factors, thus alleviating neuroinflammation (Guo et al. 2023).

The gut microbiota is essential for normal microglial maturation and function (Thion et al. 2018). One study demonstrated that microglial function in the brain was markedly impaired in germ-free mice and in antibiotic-treated mice with substantial depletion of the resident microbiota (Erny et al. 2015). As major microbial metabolites, SCFAs may enhance the anti-inflammatory properties of microglia, suppress lipopolysaccharide (LPS)-induced phagocytic activity, and modulate cytokine production in LPS-stimulated human dendritic cells (Li et al. 2021; Nastasi et al. 2015; Yamashiro et al. 2021). In the BCCAO rat model, disruption of SCFA-producing gut microbiota was accompanied by reduced serum SCFA levels. SCFA supplementation decreased the number of activated microglia in the hippocampus and suppressed the BCCAO-induced upregulation of inflammatory markers and cytokines (Xiao et al. 2022). These findings suggest that the gut microbiota may regulate microglial maturation and function through the production of SCFAs. SCFAs, in addition to affecting the anti-inflammatory function of microglia, can also directly or indirectly influence astrocyte function. Some literature confirms that SCFAs can alleviate astrocyte activation and neuroinflammation by regulating the SGK1/IL-6 signaling pathway (Gao et al. 2022). NF-κB is a eukaryotic transcription factor involved in controlling numerous normal cellular processes, including immune-inflammatory responses and microglial activation of astrocytes (Cheng et al. 2022). SCFAs, particularly sodium butyrate, inhibits NF-κB activation and reduces microglia-mediated astrocyte activation (Su et al. 2022; Luhrs et al. 2002). In conclusion, SCFAs may inhibit neuroinflammation by suppressing the inflammatory responses of both microglia and astrocytes.

SCFAs may also attenuate neuroinflammation by regulating peripheral and meningeal immune responses. Following cerebral ischemia, meningeal γδ T cells secrete IL-17 and aggravate ischemic injury, whereas regulatory T cells (Tregs) restrain this response (Lee et al. 2020; Benakis et al. 2016). Sodium butyrate promotes Treg expansion in ischemic brain regions, reduces IL-17 expression, and lowers cerebral TNF-α levels (Li et al. 2023). Moreover, SCFA-producing bacteria, including members of the genera Clostridium, Lactobacillus, and Bacteroides, may contribute to immune homeostasis by promoting Treg differentiation (Lee et al. 2021; Forbes et al. 2016). SCFAs also restore the CCH-induced imbalance between Tregs and Th17 cells, thereby alleviating intestinal barrier dysfunction and chronic inflammation (Daulatzai 2016). Collectively, these findings suggest that SCFAs may attenuate neuroinflammation by modulating immune regulatory responses.

### SCFAs preserve BBB integrity

SCFAs play a critical role in maintaining the integrity of the BBB. CCH disrupts endothelial tight junctions, whereas SCFA supplementation may restore the expression of tight junction proteins and reduce BBB permeability (Su et al. 2023). Acetate has been reported to preserve BBB function partly by modulating microglial activity (Nagpal et al. 2019). In in vitro cell experiments, propionate regulates the response and differentiation of central nervous system Treg cells, reducing oxidative stress-induced damage to the BBB and protecting its integrity (Dong and Cui 2022). Studies have demonstrated that in a mouse model of traumatic brain injury, intraperitoneal administration of sodium butyrate increased the expression of the tight junction proteins zonula occludens-1 (ZO-1) and occludin, thereby attenuating BBB disruption (Ahmed et al. 2022). IL-22 is known to maintain epithelial cell barriers and prevent inflammation-induced epithelial damage. Butyrate significantly enhances IL-22 expression in the brain, protecting BBB integrity and improving stroke prognosis (Chen et al. 2023; Wang et al. 2021). SCFAs enhance Treg expression, which in turn suppresses IL-17 production and reduces MMP-9 levels, thus strengthening BBB integrity (Park et al. 2009). As an important component of SCFAs, sodium butyrate increases GLP-1 secretion through the gut-brain axis, significantly enhancing the expression of tight junction proteins (occludin, zoonula, and occludin-1), reducing BBB damage, and improving neurological function (Li et al. 2018). In summary, these findings suggest that gut microbiota-derived SCFAs may contribute to the restoration and maintenance of BBB integrity.

### SCFAs maintain normal mitochondrial function

Disruption of mitochondrial energy metabolism is a major contributor to oxidative stress, neuronal apoptosis, and neurological dysfunction during CCH.Maintenance of mitochondrial function is essential for neuronal energy-dependent processes, including neurotransmitter synthesis, synaptic transmission, and ion homeostasis (Mayevsky et al. 2020). Gut microbiota-derived metabolites, such as SCFAs, have been shown to ameliorate ischemia-induced mitochondrial metabolic disturbances (Xiao et al. 2022). In BCCAO rats, the accumulation of ROS leads to mitochondrial dysfunction, but SCFAs increase the levels of acetate, acetyl-CoA, and ATP, normalizing mitochondrial membrane potential, reducing ROS accumulation, and restoring mitochondrial function in the hippocampus (Su et al. 2023). Another study found that SCFAs have also been reported to restore the activity of mitochondrial electron transport chain complexes and the expression of mitochondrial complex-associated proteins, resulting in improved ATP production in CCH rats (Su et al. 2022). Evidence from other neurological models further suggests that propionate may attenuate oxidative stress-induced mitochondrial swelling and vacuolization and reduce neuronal apoptosis (Cheng et al. 2019; Li et al. 2021). These studies indicate that SCFAs can mitigate oxidative stress-induced mitochondrial dysfunction, reduce ROS production, and protect mitochondrial function, thereby improving the energy metabolism disturbances caused by ischemia-hypoxia.

Mitophagy may also contribute to mitochondrial quality control after chronic cerebral ischemia, although its precise role appears to be context-dependent. Maintenance of mitochondrial function is essential for neuronal energy-dependent processes, including neurotransmitter synthesis, synaptic transmission, and ion homeostasis (Su et al. 2018; Xiao et al. 2023). In contrast, other studies have reported that mitophagic activity remains insufficient despite an apparent increase following BCCAO rats and that further induction of mitophagy with rapamycin may alleviate vascular cognitive impairment (Wang et al. 2023). These apparently divergent findings suggest that both insufficient and excessive mitophagy may be detrimental and that an appropriate level of mitophagic activity is required to maintain mitochondrial homeostasis.

### SCFAs promote synaptic plasticity and cognitive function

Impaired synaptic plasticity and abnormal synaptic vesicle release are important neuropathological features associated with cognitive dysfunction after cerebral ischemia (Sadler et al. 2020; Yan et al. 2021). Increasing evidence suggests that SCFAs may preserve synaptic integrity and cognitive function through epigenetic regulation and neurotrophic signaling.Studies have shown that in mice with a dietary fiber deficiency, hippocampal synaptic structures are damaged, and calcium/calmodulin-dependent protein kinase II (CaMKII) activity is abnormal, leading to cognitive dysfunction. SCFA supplementation partially restored synaptic protein expression and improved hippocampal synaptic structure in these animals (Shi et al. 2021). Histone deacetylases (HDACs) play significant roles in central nervous system diseases. For example, HDAC3 is a key negative regulator of long-term memory formation, and the expression level of HDAC4 is significantly associated with the improvement of neuronal remodeling (Wu et al. 2013; Yu et al. 2020). SCFAs may also regulate synaptic plasticity through the inhibition HDACs.For example,Acetate has been reported to improve hippocampal synaptic plasticity in mouse models of depression through HDAC inhibition (Huang et al. 2021). As an HDAC inhibitor, sodium butyrate also improved synaptic plasticity and attenuated early neuroinflammation in 5XFAD mice (Jiang et al. 2021). Brain-derived neurotrophic factor (BDNF) is essential for neurogenesis, synaptic plasticity, and neuronal survival. SCFAs may enhance hippocampal BDNF expression and thereby modulate cognitive-related behaviors (Church et al. 2023). In a neonatal rat hypoxia–ischemia model, sodium butyrate stimulated neurogenesis in the damaged ipsilateral side via the BDNF-TrkB signaling pathway and restored the number of neuronal cells in the ipsilateral subventricular zone (Jaworska et al. 2019). This suggests that sodium butyrate can promote the recovery of neurological function in ischemia-hypoxia rats. In summary, SCFAs can influence synaptic plasticity through multiple mechanisms, thereby improving cognitive dysfunction caused by CCH and providing a theoretical basis for the clinical treatment of VaD following CCH.

### Potential adverse effects of SCFAs

Despite their generally beneficial effects on intestinal barrier integrity, immune homeostasis, and neuroinflammation, SCFAs should not be considered universally protective, as their biological effects vary according to concentration, tissue type, cellular metabolic state, and disease context. At high intracellular concentrations, butyrate functions as a HDAC inhibitor and may alter the expression of genes involved in cell-cycle regulation, differentiation, proliferation, and apoptosis (Donohoe et al. 2012). Butyrate-mediated HDAC inhibition has also been reported to suppress type I interferon responses by reducing the expression of antiviral proteins, including RIG-I and IFITM3, thereby increasing susceptibility to viral infection in cultured human and murine cells (Chemudupati et al. 2020). Excessive butyrate exposure may also interfere with tissue regeneration. Following colonic mucosal injury, direct exposure of intestinal stem and progenitor cells to butyrate may suppress their proliferation through an HDAC- and Foxo3-dependent mechanism, potentially delaying epithelial repair (Kaiko et al. 2016). Propionate has likewise been associated with adverse metabolic effects under certain experimental conditions.Propionate may enhance catecholamine-mediated counter-regulatory signaling, thereby contributing to insulin resistance and compensatory hyperinsulinemia, which may promote obesity and related metabolic disturbances (Tirosh et al. 2019). Nevertheless, most of these potential adverse effects have been identified in cell-based studies, animal models, or short-term metabolic experiments, and their clinical relevance to patients with CCH remains uncertain. Future clinical studies should therefore define the therapeutic window for individual SCFAs and systematically evaluate the effects of dose, formulation, route of administration, treatment duration, host genetic and metabolic characteristics, immune status, susceptibility to infection, and so on.

## Strategies for modulating the gut–brain axis: advantages and disadvantages

### Fecal microbiota transplantation (FMT)

Fecal microbiota transplantation (FMT) has emerged as a potential microbiota-targeted strategy for promoting recovery after CCH. By partially restoring gut microbial homeostasis and modulating immune responses associated with post-ischemic dysbiosis, FMT may improve neurological outcomes after stroke (Singh et al. 2016; Panther et al. 2022). Ischemic stroke is associated with reduced intestinal levels of SCFAs, suggesting that restoration of SCFA-producing microbiota may represent a potential therapeutic strategy. FMT enriched with SCFA-producing bacteria has been shown to alleviate gut dysbiosis and intestinal barrier dysfunction, as well as reduce neuronal injury in experimental models of CCH (Chidambaram et al. 2022; Shi et al. 2021). In addition, FMT may improve mitochondrial function by enhancing electron transport chain activity and oxidative phosphorylation in both colonic and brain tissues, thereby partially restoring mitochondrial energy metabolism (Su et al. 2022; Liu et al. 2020). This indicates that FMT could serve as a potential therapeutic target to mitigate mitochondrial energy metabolism disorders induced by CCH in colonic and brain tissues. In summary, FMT may attenuate inflammation, preserve intestinal and blood–brain barrier integrity, and improve mitochondrial function in preclinical models of CCH.

However, current research on FMT is still at the animal experimentation stage and has not yet been applied clinically. In rodent models, FMT alleviates gut dysbiosis, neuroinflammation, and cognitive impairment.Whereas,its efficacy and safety in humans remain uncertain (Xiao et al. 2022; Rees et al. 2022). Clinical translation is constrained by donor selection, safety, standardization, and ethical concerns. Donors require rigorous clinical, blood, and stool screening to reduce the risk of transmitting pathogens or other harmful microbiome-associated traits (Cammarota et al. 2019; Rondinella et al. 2024). Reported adverse events, including gastrointestinal discomfort and fever, further highlight the need for systematic safety assessment (Wang et al. 2016). Future studies should establish standardized donor-screening and treatment protocols and evaluate the long-term safety and efficacy of FMT in well-designed clinical trials. With improved standardization and microbiome-based patient stratification, FMT may become a promising individualized intervention for CCH-related cognitive impairment.

### Application of probiotics and prebiotics

Prebiotics are defined as “substrates that are selectively utilized by host microorganisms and confer health benefits” (Yadav et al. 2022). Probiotics are defined by the World Health Organization as live microorganisms that, when administered in adequate amounts, confer a health benefit to the host (Clottes et al. 2023). Both probiotics and prebiotics may exert beneficial effects by modulating the gut microbial community, promoting the production of SCFAs, regulating immune responses, and preserving intestinal barrier integrity (Sanders et al. 2019).

In patients with depression, prebiotic supplementation has also been associated with alterations in gut microbial β-diversity, improvements in microbial composition, and reductions in depressive symptoms (Alli et al. 2022). Moreover, reduced production of gut microbiota-derived short-chain fatty acids (SCFAs) in older adults is associated with post-stroke immune dysregulation and unfavorable outcomes. Supplementation with SCFA-producing bacteria or dietary substrates that promote SCFA production may help restore SCFA levels and improve stroke outcomes (Lee et al. 2020). The combined administration of probiotics and prebiotics is commonly referred to as synbiotic supplementation (Sorbara and Pamer 2022). A randomized controlled trial demonstrated that synbiotic supplementation increased serum brain-derived neurotrophic factor (BDNF) levels and alleviated depressive symptoms compared with the control intervention (Haghighat et al. 2021). Similarly, another clinical study reported a greater reduction in depression scores among patients receiving synbiotics than among those receiving placebo (Ghorbani et al. 2018). However, research on the use of synbiotics for depression remains limited, and the available evidence has focused primarily on clinical efficacy. Basic research investigating the potential mechanisms of action is still insufficient, and the underlying biological mechanisms require further investigation and validation. In summary, prebiotics, probiotics and Synbiotic can affect the gut microbiota, maintain gut barrier function, and improve dysfunction associated with chronic cerebral ischemia through the gut-brain axis.

However, the translation of prebiotic and probiotic interventions into human clinical practice remains challenging. First, different probiotic strains have distinct functions and mechanisms of action, and their effects may vary depending on individual differences (McFarland et al. 2018; Zmora et al. 2018). Therefore, the specificity of the strains and individual adaptability should be considered when selecting prebiotics and probiotics. Second, Although probiotics and prebiotics are generally well tolerated, transient gastrointestinal symptoms, including gas, bloating, and abdominal discomfort, may occur, particularly with highly fermentable prebiotics or higher doses (Ciorba 2012). Third, The biological effects of probiotics and prebiotics involve complex interactions among the gut microbiota, microbial metabolites, intestinal-barrier function, immune responses, and host metabolic pathways (Markowiak and Slizewska 2017). Nevertheless, the precise molecular mechanisms underlying many of these effects remain incompletely understood. Further mechanistic and well-designed clinical studies are therefore required to clarify strain- or substrate-specific effects, identify determinants of individual responsiveness, and support their rational and evidence-based application.

### Supplementation of SCFAs

SCFAs may contribute to cognitive and neurological recovery following CCH through both central and peripheral mechanisms. Acetate and propionate, as the major components of SCFAs, have been shown to easily cross the BBB and influence brain function in development, health, and disease (Frost et al. 2014). In addition, butyrate supplementation has been shown to modulate the gut microbiota and exert neuroprotective effects in experimental ischemic stroke. These effects include reductions in neuronal injury, cerebral infarct volume, and brain edema (Wu et al. 2013).

The neuroprotective effects of SCFAs appear to be mediated, at least in part, by the suppression of neuroinflammation. SCFA supplementation has been reported to inhibit the activation of microglia and astrocytes and to promote a shift in microglial polarization from a pro-inflammatory M1 phenotype toward an anti-inflammatory M2 phenotype (Nastasi et al. 2015). In CCH models, SCFA supplementation also reduces the inflammatory response of astrocytes, alleviating neurological damage (Maslowski et al. 2009). Oral supplementation of SCFAs can directly promote neurological recovery by crossing the BBB, and can also indirectly influence neurological function by improving the gut microbiota, thus promoting cognitive recovery after CCH (Silva et al. 2020). These findings suggest that SCFAs may alleviate CCH-induced neurological damage by modulating glial activation and limiting the production of pro-inflammatory mediators.

However, no clinical studies have systematically evaluated direct SCFA supplementation in patients with CCH, and the optimal composition, dose, route of administration, timing of initiation, and treatment duration remain unknown. In BCCAO rat models, a mixture of sodium acetate(67.5 mM), sodium propionate(25.9 mM), and sodium butyrate(40 mM) has been administered continuously after the induction of hypoperfusion for periods ranging from 14 days to approximately 5 weeks, with early reductions in neuroinflammation and subsequent improvements in neuronal survival and cognitive and depressive-like behaviors (Xiao et al. 2022; Zou et al. 2018). In addition, sodium butyrate(840 mg/kg/d) administered for 4 weeks, beginning 29 days after arterial occlusion, improved cognitive impairment in a 2VO rat model (Liu et al. 2015). Nevertheless, these animal regimens cannot be directly extrapolated to humans. The optimal SCFA composition, dose, route of administration, treatment initiation, and duration should therefore be determined in future dose-ranging clinical studies with pharmacokinetic, pharmacodynamic, efficacy, and safety assessments.

### Animal experiments

Animal studies currently provide the principal evidence supporting the efficacy of FMT, probiotics and prebiotics, and direct SCFA supplementation. In mice with spinal cord injury, FMT increased SCFA levels and promoted functional recovery and neuronal regeneration (Jing et al. 2021). In rats with CCH, transplantation of gut microbiota from healthy donors increased SCFA concentrations, attenuated intestinal and hippocampal inflammation, and reduced hippocampal neuronal apoptosis, as indicated by an increased Bcl-2/Bax ratio and elevated Bcl-xL expression (Xiao et al. 2022). In related preclinical models, FMT-induced increases in acetate were accompanied by restoration of the Treg/Th17 balance and reduced production of inflammatory mediators (Su et al. 2022). Together, these findings indicate that FMT exerts broad effects on microbial composition, metabolite production, immune regulation, and tissue energy metabolism.

Evidence for probiotics and prebiotics also supports an indirect microbiota-dependent mechanism. Fructooligosaccharide supplementation altered the gut microbial community, increased the abundance of Prevotella, and elevated cecal concentrations of acetate, butyrate, and valerate (Cui et al. 2022). These findings indicate that the effects of probiotics and prebiotics may depend on the specific strain or substrate used, as well as on the capacity of the recipient microbiota to metabolize these interventions and generate beneficial metabolites.

Direct SCFA supplementation has likewise shown neuroprotective effects in experimental models of CCH. In CCH research, SCFA supplementation alleviated neuronal loss and microglia-mediated synaptic loss, maintaining the normal processes of synaptic vesicle fusion and release, thereby enhancing synaptic plasticity (Nastasi et al. 2015). Across these preclinical studies, attenuation of pro-inflammatory signaling, inhibition of neuronal apoptosis, and preservation of neuronal and synaptic function emerged as common downstream effects.

## Future perspectives

Current evidence supporting microbiota-targeted and short-chain fatty acid (SCFA)-based interventions is derived predominantly from animal studies, whereas relevant clinical evidence remains limited. Although fecal microbiota transplantation, probiotics, prebiotics, and direct SCFA supplementation have demonstrated potential neuroprotective effects in animal models of chronic cerebral hypoperfusion (CCH), their clinical efficacy, safety, and long-term benefits have not yet been adequately established.

Future studies should further elucidate the molecular and cellular mechanisms through which microbiota-targeted interventions and SCFA-based therapies exert neuroprotective effects. Particular attention should be given to establishing causal relationships among gut microbial composition, SCFA metabolism, immune regulation, blood–brain barrier integrity, and neural plasticity. Well-designed clinical studies with adequate sample sizes are also required to systematically evaluate the efficacy, safety, and interindividual variability of these interventions in patients with CCH. Because treatment responses may be influenced by baseline gut microbiota composition, dietary patterns, age, comorbidities, and concomitant medications, future research should identify microbial, metabolic, and inflammatory biomarkers that can predict therapeutic responsiveness and facilitate patient stratification and personalized intervention.

In addition, the formulation, composition, dosage, route of administration, timing of treatment initiation, and intervention duration require further optimization, together with the establishment of standardized treatment protocols and outcome measures. Future efforts should focus on developing microbial preparations, dietary substrates, and pharmacological agents with well-defined targets, reproducible biological effects, and favorable translational potential. Integration of basic research, translational medicine, and clinical trials may ultimately facilitate the development of more precise, safe, and effective therapeutic strategies for cognitive impairment associated with CCH.

## Conclusion

In summary, CCH induces a broad spectrum of pathological alterations, including neuroinflammation, disruption of the BBB, mitochondrial dysfunction, and synaptic impairment. In recent years, the MGBA has emerged as an important area of research in this field. CCH may induce gut microbial dysbiosis accompanied by reduced production of SCFAs. SCFAs play important roles in the development and maintenance of central nervous system homeostasis and may contribute to the regulation of cognitive function after CCH through the GMBA. Accumulating evidence suggests that SCFAs may attenuate CCH-induced neurological injury and cognitive impairment through multiple mechanisms, including suppression of neuroinflammation, preservation of BBB integrity, improvement of mitochondrial function, and enhancement of synaptic plasticity. In animal models, both direct SCFA supplementation and strategies that indirectly increase SCFA levels, such as FMT, probiotic or prebiotic supplementation, have demonstrated potential neuroprotective effects. Future studies should further evaluate the efficacy and safety of microbiota-targeted and SCFA-based interventions in patients with CCH through rigorously designed clinical trials and optimize their administration strategies, dosages, and treatment durations to facilitate clinical translation.

## Data Availability

No datasets were generated or analysed during the current study.
